# Effectiveness of Extracorporeal Cardiopulmonary Resuscitation for Non-Shockable Out-of-Hospital Cardiac Arrest: An Analysis From the SOS-KANTO 2017 Study

**DOI:** 10.7759/cureus.69305

**Published:** 2024-09-13

**Authors:** Toru Yoshida, Minoru Yoshida, Yoshihiro Masui, Takaki Naito, Jumpei Tsukuda, Kenichiro Morisawa, Shigeki Fujitani

**Affiliations:** 1 Department of Emergency and Critical Care Medicine, St. Marianna University School of Medicine, Kawasaki, JPN

**Keywords:** ecmo, e-cpr, neurological outcome, non-shockable ohca, survival rate

## Abstract

Extracorporeal cardiopulmonary resuscitation (ECPR) using veno-arterial extracorporeal membrane oxygenation (V-A ECMO) has the potential as a viable treatment for refractory out-of-hospital cardiac arrest (OHCA). While mechanical circulatory support devices, such as Impella® and left ventricular assist devices, are being increasingly used, initial ECPR often relies on V-A ECMO. Previous studies, including randomized controlled trials, reported the prognostic benefits of ECPR for shockable OHCA (SOHCA); however, its effectiveness for non-SOHCA (NSOHCA) remains unclear, with poorer neurological outcomes and the lower return of spontaneous circulation rates than for SOHCA being reported. The present study utilized data from the SOS-KANTO 2017 study to examine the impact of ECPR on the neurological outcomes of NSOHCA. Data from 2,502 OHCA cases were analyzed, with a focus on the relationship between ECPR and 90-day neurological outcomes. The results obtained showed significantly higher survival rates at 30 and 90 days and significantly better 90-day neurological outcomes in the ECMO attempt group than in the non-ECMO attempt group. A multivariate analysis identified ECPR as one of the significant independent predictors of favorable neurological outcomes. The prognosis of NSOHCA cases with CA was improved by ECPR using V-A ECMO, particularly in those where CPR was initiated within one minute of onset and the patient arrived at the hospital within 45 minutes. Factors associated with a favorable prognosis included a shorter time from onset to hospital arrival and the likelihood of acute coronary syndrome being the cause of CA. The present results suggest the potential of ECPR to improve the survival and the 90-day prognosis of NSOHCA, particularly when bystander CPR is initiated quickly and hospital arrival is prompt.

## Introduction

Extracorporeal cardiopulmonary resuscitation (ECPR) using veno-arterial extracorporeal membrane oxygenation (V-A ECMO) is performed for treatment-resistant out-of-hospital cardiac arrest (OHCA). Mechanical circulatory support devices, such as micro-axial pump catheters (Impella®) and left ventricular assist devices, are also more commonly used; however, the initial stages of ECPR often involve the use of V-A ECMO. Regarding the effects of ECMO on cases of CA, previous studies, including randomized controlled trials, reported that the prognosis of OHCA, particularly shockable OHCA (SOHCA), was improved by ECPR [1-3]. However, in some studies that included non-shockable OHCA (NSOHCA), such as pulseless electrical activity and asystole (A-sys), no significant differences were observed in neurological outcomes [4,5]. Furthermore, NSOHCA had a lower likelihood of achieving the return of spontaneous circulation (ROSC) and a worse prognosis than SOHCA [6,7]. Therefore, NSOHCA is less likely to be associated with the adoption of aggressive CPR, particularly ECPR, which involves significant costs and effort.

In contrast, in the SOS-KANTO 2012 study, a registry of OHCA in the Kanto region, we demonstrated that among NSOHCA cases, there was a subgroup in which the underlying cause of arrest correlated with a favorable prognosis [6]. Within this study, we also identified a subgroup where the use of ECPR was shown to improve the prognosis [8]. Observational studies [9-13] reported setting the goal of initiating ECMO within 60 minutes of an emergency call in order to achieve a favorable prognosis, and at least 15 minutes was assumed to be required from hospital arrival to the initiation of V-A ECMO.

In the present study, using data from the SOS-KANTO 2017 study, we examined the relationship between the implementation of ECPR at the time of emergency medical services (EMS) contact in NSOHCA and neurological outcomes.

Part of the contents of this article was previously presented as a meeting abstract at the 88th Annual Scientific Meeting of the Japanese Circulation Society on March 10, 2024.

## Materials and methods

Study design

We analyzed data from patients enrolled in the SOS-KANTO 2017 study using a methodology similar to that employed in the SOS-KANTO 2012 study [8,14-16]. Among these patients, a prospective survey was conducted to collect pre- and in-hospital as well as follow-up data on patients with CA who were admitted to 42 emergency hospitals (Appendix A, Appendix B) across the Kanto region (central Japan) between September 9, 2019, and March 8, 2021, using a methodology similar to that employed in the SOS-KANTO 2012 study [8,14-16]. The present study is a retrospective observational study using data collected from the SOS-KANTO 2017 and was approved by the Ethics Committee of the authors’ institution (approval number 4451) as well as the Ethics Committees of 41 other institutions. The requirement for informed patient consent was waived to ensure participant anonymity, in accordance with guidelines issued by the Japanese government, using a methodology similar to that employed in the SOS-KANTO 2012 study [8,14-16].

Participants

**Figure 1:**
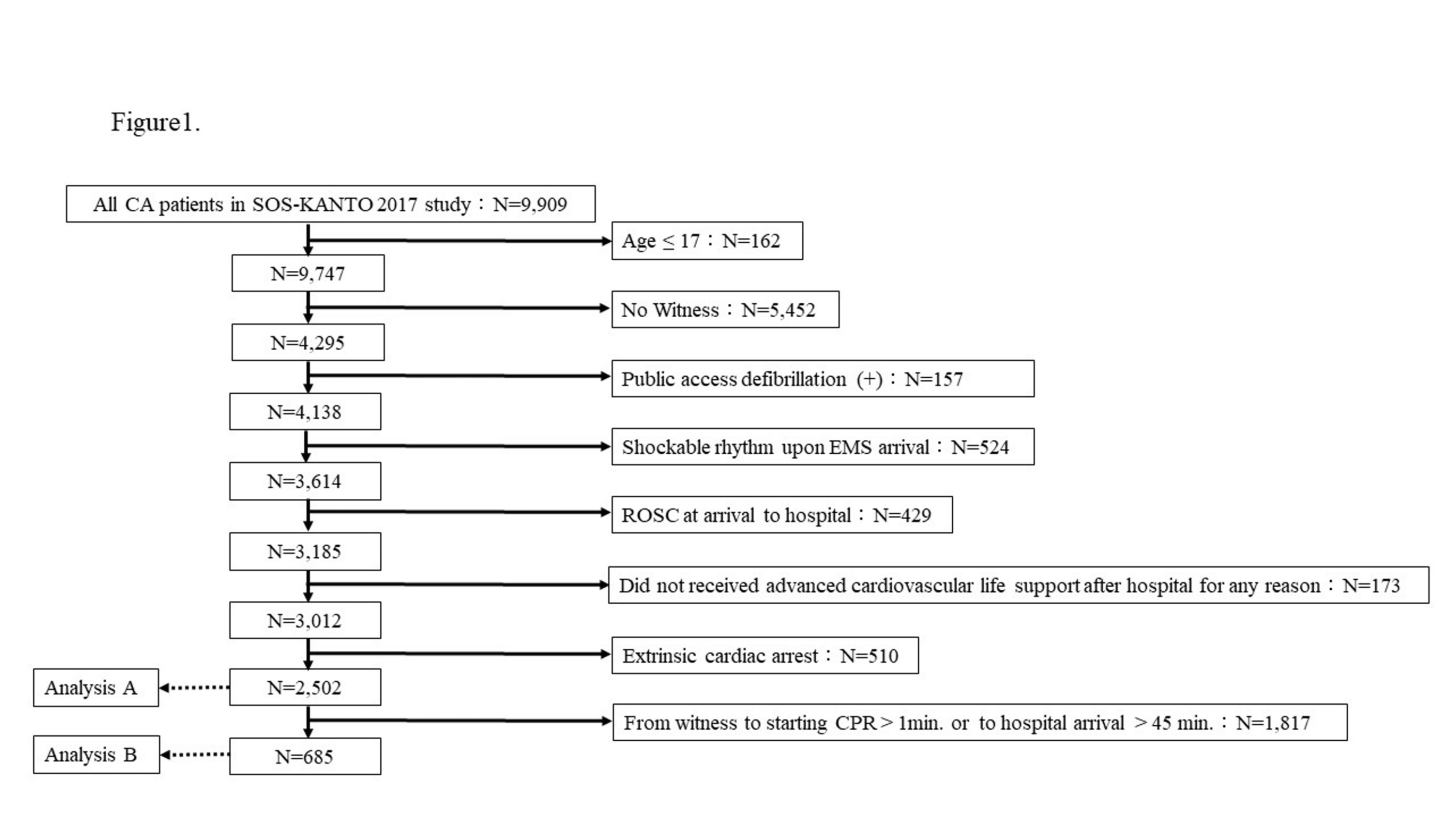
Flowchart of the patient selection process Patients younger than 18 years, CAs that were unwitnessed, patients on whom defibrillation was performed by laypeople, patients who were in a shockable rhythm of OHCA at the time of EMS contact or developed CA with a shockable rhythm after EMS contact, patients who had achieved ROSC at arrival to the hospital, patients who did not receive advanced cardiovascular life support for any reason, and CAs due to external causes were excluded from Analysis A. Patients with >1-minute interval from witnessing CA to initiating CPR or >45 minutes to arrival at hospital were excluded from Analysis B. After applying these exclusion criteria, 7,407 of the initial 9,909 patients were excluded, and the remaining 2,502 were included in Analysis A, while 9,224 patients were excluded and the remaining 685 were included in Analysis B. CA, cardiac arrest; CPR, cardiopulmonary resuscitation; EMS, emergency medical services; OHCA, out-of-hospital cardiac arrest; ROSC, return of spontaneous circulation

A total of 9,909 cases of OHCA were registered in the SOS-KANTO 2017 study. Exclusion criteria were as follows: (1) patients younger than 18 years; (2) unwitnessed CA; (3) patients on whom defibrillation was performed by laypeople; (4) patients who were in a shockable rhythm of OHCA at the time of EMS contact or who developed CA with a shockable rhythm after EMS contact; (5) patients who had achieved ROSC at arrival to the hospital; (6) patients who did not receive advanced cardiovascular life support for any reason; and (7) CA due to external causes. This process identified 2,502 cases that were eligible for the present study (Figure 1). Analysis A primarily focused on examining the relationship between clinical conditions, including the administration or absence of ECPR, and neurological outcomes 90 days after the event, classified as a cerebral performance category (CPC) of 1 to 2.

Analysis B involved a specific analysis of 685 cases in which CPR was initiated within one minute of witnessing CA and arrival at the hospital occurred within 45 minutes of CA being witnessed (Figure 1). The rationale behind this analysis was based on the prompt initiation of bystander CPR [17], as the importance of early initiation of CPR was emphasized, but the definition of “prompt” was unclear. Therefore, in this study, we defined prompt bystander CPR as that initiated within one minute of witnessing the event. Additionally, the initiation of ECPR within one hour from the onset of CA is crucial for improving outcomes [9]. Furthermore, Sakamoto et al. reported improved outcomes with ECPR in their study, where the inclusion criteria specified that patients either arrived at the hospital within 45 minutes of CA onset or the 119 emergency call and did not achieve ROSC within 15 minutes after hospital arrival [2]. Based on this, approximately 15 minutes was assumed to elapse from hospital arrival to the commencement of ECPR, which guided this focused approach.

Definitions and data collection

We defined CA as the cessation of cardiac mechanical activity using a methodology similar to that employed in the SOS-KANTO 2012 study, namely, the absence of a detectable pulse, responsiveness, and normal breathing [8,18,19]. All CA patients who were transported to the participating hospitals by EMS providers were included in the SOS-KANTO 2017 study. Pre- and in-hospital treatments were generally delivered by EMS personnel, physicians, and other health care providers in accordance with the national guidelines of the Japan Resuscitation Council based on international guidelines using a methodology similar to that employed in the SOS-KANTO 2012 study [8,14-16,20]. EMS providers collected prehospital information according to the standardized Utstein style [15], including patient sex and age, the initial cardiac rhythm, and the resuscitation timeline (e.g., the time at which CPR was initiated by a bystander or EMS provider, the presence or absence of VF or pulseless VT, whether EMS provided defibrillation, and the time of hospital arrival). The following information was also included: whether CA was witnessed by a bystander, when and whether CPR was performed by a bystander or EMS personnel, and when the patient arrived at the hospital. It was not possible to assess the quality of CPR using a methodology similar to that employed in the SOS-KANTO 2012 study [8,14-16]. We evaluated neurological outcomes and reported them using the CPC scale scored as follows: category 1, good cerebral performance; category 2, moderate cerebral disability; category 3, severe cerebral disability; category 4, coma or a vegetative state; and category 5, brain death or patient death using a methodology similar to that employed in the SOS-KANTO 2012 study [8,18,19,21].

Analysis

We planned two different analyses - Analysis A and Analysis B - with different criteria for inclusion as discussed. Analysis B specifically targeted cases in which CPR was initiated within one minute and hospital arrival was within 45 minutes from witnessing CA.

In each analysis, we divided participants into two groups based on whether ECMO was attempted after arrival. We compared background factors upon arrival, underlying diseases, and outcomes. In the ECMO attempt group, we evaluated the interval from arrival until the start of ECMO. We compared background factors upon arrival, such as age, sex, the interval from the onset of CA until arrival, and underlying diseases. Regarding outcomes, we compared 30- and 90-day survival rates (CPC 1-4) and favorable cerebral function rates at 30 and 90 days (CPC 1-2). Concerning survival rates (CPC 1-4), we examined the effects of ECMO using Kaplan-Meier survival curves. Regarding 30- and 90-day survival rates (CPC 1-4) and cerebral function rates at 30 and 90 days (CPC 1-2), we incorporated background factors upon arrival and underlying diseases that significantly differed between the ECMO and non-ECMO attempt groups in a univariate analysis and in a multivariate logistic regression model. The analytical methods described above were conducted using a similar approach to that employed in SOS-KANTO 2012 [8].

Statistical analysis

We used JMP17 software (SAS Institute Inc., Cary, NC, United States) for statistical analyses. We expressed continuous variables as medians (IQR) and used the chi-squared test for discrete variables, except when the sample size was small, in which case we used Fisher’s exact test. Wilcoxon’s signed-rank test was used to compare continuous variables. A multivariate analysis was performed with neurological outcomes at 30 and 90 days as the dependent variable and the presence or absence of an ECMO attempt and clinical background factors as explanatory variables. We set the significance of differences to p < 0.05. These statistical methods were conducted using a similar approach to that employed in SOS-KANTO 2012 [8].

## Results

Analysis A

The present study included 2,502 cases with a median age of 77.0 years (67.5-85.0), of which 1,515 (60.6％) were male and 987 (39.4%) were female. The time from witnessing CA to hospital arrival was 37.0 minutes [28.0-47.0]. ECMO was attempted in 101 cases (4.0%) after hospital arrival (ECMO attempt group). As shown in Table 1, no significant difference was observed in the administration of adrenaline by EMS between the ECMO and non-ECMO attempt groups. Similarly, no significant difference was noted in the rate of tracheal intubation. However, the ECMO attempt group was significantly younger, had a higher percentage of males, and had a shorter time from witnessing CA to hospital arrival. Furthermore, the etiology of CA (cardiac vs. non-cardiac) did not significantly differ between the groups. However, among cardiac etiologies, acute coronary syndrome (ACS) was significantly more common in the ECMO attempt group than in the non-ECMO attempt group, whereas presumed cardiac etiology was significantly less common. Regarding non-cardiac etiologies, malignancies were significantly less common in the ECMO attempt group.

**Table 1:** Characteristics of the ECMO attempt group and the non-ECMO attempt group in Analysis A ACS: acute coronary syndrome; CPC: cerebral performance category; CPR: cardiopulmonary resuscitation; ECMO: extracorporeal membrane oxygenation; PCF: presumed cardiac factor

	Total (N = 2,502)	ECMO attempted group (N = 101)	Non-ECMO attempted group (N = 2,401)	p
Age (years), median [IQR]	77.0 [67.5-85.0]	59.0 [48.0-69.0]	78.0 [68.0-85.0]	<0.0001
Sex (Male) (N [%])	1,515 [60.6]	78 [77.2]	1,437 [59.9]	0.0005
Sex (Female) (N [%])	987 [39.4]	23 [22.8]	964 [40.1]	
Time from a witness of CA until the start of CPR [min], median [IQR]	6.0 [0.0-13.0]	3.0 [0.0-13.0]	6.0 [0.0-13.0]	0.0696
Time from beginning of CPR until hospital arrival [min], median [IQR]	29.0 [21.0-36.0]	24.0 [16.0-29.0]	29.0 [21.0-36.0]	0.0001
Time from a witness of CA until hospital arrival [min], median [IQR]	37.0 [28.0-47.0]	31.5 [21.3-39.0]	38.0 [28.0-47.0]	<0.0001
CPR performed by a bystander (N [%])	1,257 [50.2]	64 [63.4]	1,193 [49.7]	0.0071
Adrenaline administered by EMS (N [%])	951 [38.0]	43 [42.6]	908 [37.8]	0.3347
Tracheal intubation performed by EMS (N [%])	140 [5.6]	9 [8.9]	131 [5.5]	0.178
ECMO attempted (N [%])	101 [4.0]	101 [100.0]	0 [0.0]	NA
ECMO performed (N [%])	99 [4.0]	99 [98.0]	0 [0.0]	NA
Time from hospital arrival until the start of ECMO [min]	29.5 [21.8-45.5]	29.5 [21.8-45.5]	NA	NA
Etiological factors for intrinsic CA				
Cardiac factors (N [%])	1,730 [69.1]	78 [77.2]	1,652 [68.8]	0.0726
ACS (N [%])	146 [5.6]	32 [31.7]	114 [4.75]	<0.0001
Non-ACS cardiac disease (N [%])	267 [10.7]	24 [23.8]	243 [10.1]	<0.0001
PCF (N [%])	1,316 [52.6]	22 [21.8]	1,294 [53.9]	<0.0001
Noncardiac factors (N [%])	772 [30.9]	23 [22.8]	749 [31.2]	0.0726
Respiratory disease (N [%])	195 [7.8]	4 [3.96]	191 [8.0]	0.183
Cerebrovascular disease (N [%])	93 [3.7]	5 [5.0]	88 [3.7]	0.4321
Malignancy (N [%])	87 [3.5]	0 [0.0]	87 [3.6]	0.0482
Other noncardiac disease (N [%])	397 [15.9]	14 [13.9]	383 [16.0]	0.677
30 days survival (N [%])	66 [2.7]	10[10.0]	56 [2.3]	0.0002
CPC = 1; 2 after 30 days (N [%])	20 [0.8]	2 [2.0]	18 [0.8]	0.1888
90 days survival (N [%])	62 [2.5]	6 [6.0]	31 [1.3]	<0.0001
CPC = 1; 2 after 90 days (N [%])	19 [0.8]	4 [4.0]	15 [0.6]	0.006

**Figure 2:**
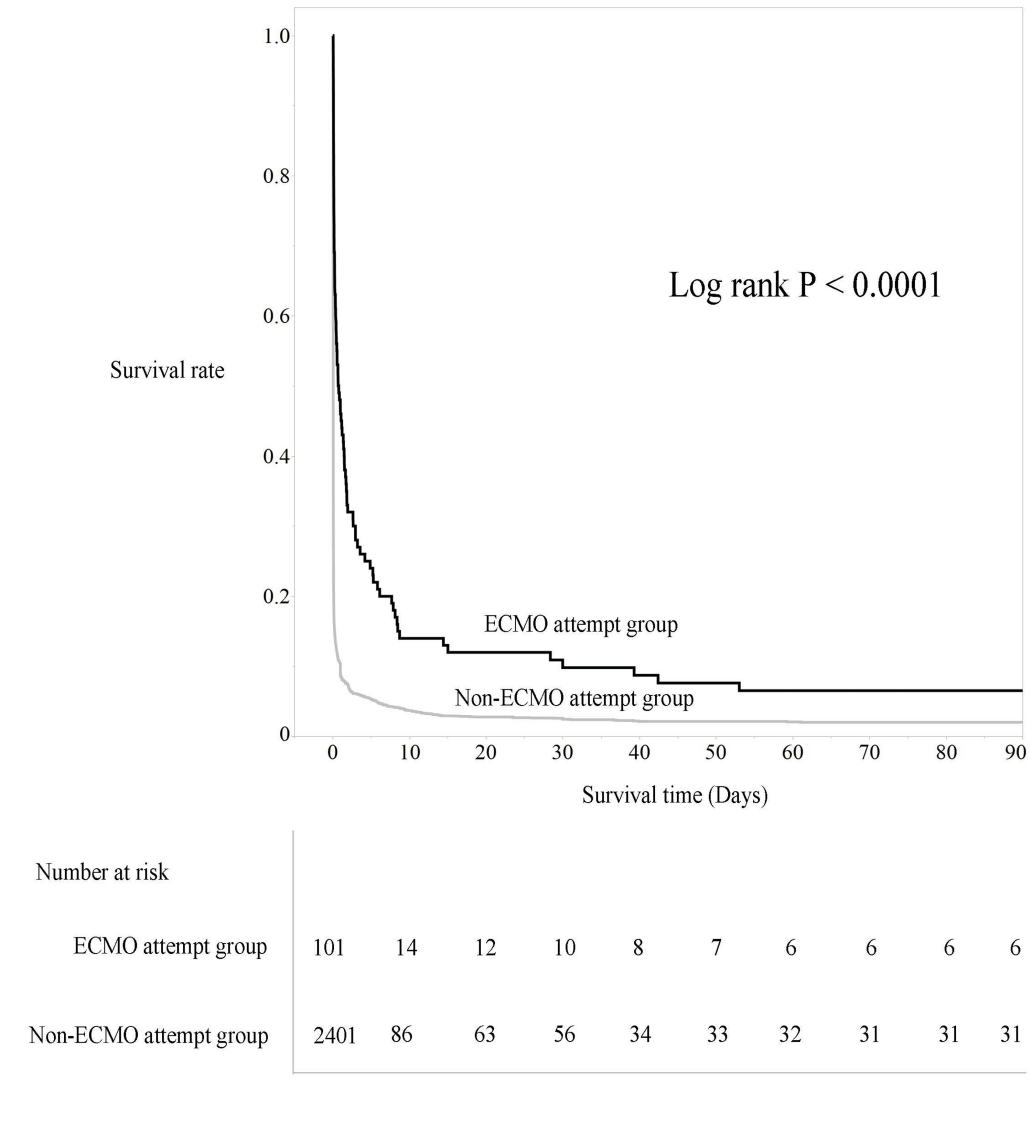
Comparison of survival rates based on the Kaplan-Meier curve in Analysis A A significant difference (p < 0.0001) was observed in the survival rate between the ECMO attempt group (black line) and the non-ECMO attempt group (grey line) from the early phase. The cumulative survival rate at 90 days was significantly higher in the ECMO attempt group. Vertical axis: survival rate (%); horizontal axis: survival period (days); blue line: the ECMO attempt group; red line: the non-ECMO attempt group ECMO: extracorporeal membrane oxygenation

Regarding survival rates, as shown in Figure 2, Kaplan-Meier survival curves indicated significantly longer survival up to 90 days in the ECMO attempt group. Regarding favorable neurological outcomes, as shown in Table 1, the percentage of patients with CPC 1-2 at 30 days was 2.0% in the ECMO attempt group and 0.8% in the non-ECMO attempt group, with no significant difference. However, at 90 days, the percentage of patients with CPC 1-2 was significantly higher in the ECMO attempt group.

Additionally, a multivariate model was created to examine favorable neurological outcomes (CPC 1-2) with an ECMO attempt and various explanatory variables. However, since there were only 19 cases with favorable neurological outcomes, only two explanatory variables could be included in a single multivariate model-whether an ECMO attempt was made and one other variable. Therefore, we combined each explanatory variable listed in Table 1 with the ECMO attempt and selected three models that were both medically plausible and had the highest R-squared values. As shown in Table 2, the combination of an ECMO attempt and the time from witnessing CA to hospital arrival had the highest R squared (0.2201) and was considered the best predictive model for favorable neurological outcomes (CPC 1-2). In this context, an ECMO attempt was an independent prognostic factor. However, in a model including ACS, the R squared was 0.055, indicating a poorer fit, and an ECMO attempt and ACS were independent prognostic factors.

**Table 2:** Multivariate analyses: favorable cerebral function outcome (CPC = 1/2) at 90 days in Analysis A ACS: acute coronary syndrome; CPC: cerebral performance category; ECMO: extracorporeal membrane oxygenation; PCF: presumed cardiac factor

	OR	95% CI	p	R squared
ECMO attempted	4.567	1.356-15.37	<0.0001	0.2201
Time from a witness of CA until hospital arrival (Δ = 1 min)	0.998	0.998-0.999	0.0294	
ECMO attempted	3.883	1.257-12.00	0.0184	0.1493
PCF	<0.001	NA	0.981	
ECMO attempted	3.829	1.088-13.48	0.0365	0.055
ACS	4.107	1.287-13.10	0.017	

Analysis B

The study included 685 cases with a median age of 76.0 years [66.0-85.0], of which 429 (62.6%) were male and 256 (37.4%) were female. ECMO was attempted in 37 cases (5.4%) after hospital arrival (ECMO attempt group). As shown in Table 3, there was no significant difference in the administration of adrenaline by EMS between the ECMO and non-ECMO attempt groups. Similarly, no significant difference was observed in the rate of tracheal intubation. However, the ECMO attempt group was significantly younger, although there was no significant difference in sex. There was also no significant difference between the groups regarding the etiology of CA (cardiac vs. non-cardiac). However, among cardiac etiologies, ACS was significantly more common in the ECMO attempt group, while presumed cardiac etiology was significantly less common. Furthermore, there were no significant differences in non-cardiac etiologies.

**Table 3:** Characteristics of the ECMO attempt group and the non-ECMO attempt group in Analysis B ACS: acute coronary syndrome; CPC: cerebral performance category; CPR: cardiopulmonary resuscitation; ECMO: extracorporeal membrane oxygenation; PCF: presumed cardiac factor

	Total (N = 685)	ECMO attempted group (N = 37)	Non-ECMO attempted group (N = 64)	p
Age (years), median [IQR]	76.0 [66.0-85.0]	62.0 [48.0-71.0]	77.0 [67.0-85.8]	<0.0001
Sex (Male) (N [%])	429 [62.6]	28 [75.6]	401 [61.9]	0.0917
Sex (Female) (N [%])	256 [37.4]	9 [24.3]	247 [38.1]	
Time from a witness of CA until the start of CPR [min], median [IQR]	0.0 [0.0-0.0]	0.0 [0.0-0.0]	0.0 [0.0-0.0]	0.3536
Time from beginning of CPR until hospital arrival [min], median [IQR]	25.0 [16.0-34.0]	21.5 [11.0-28.0]	26.0 [16.0-34.0]	0.0447
Time from a witness of CA until hospital arrival [min], median [IQR]	26.0 [16.0-34.0]	23.0 [11.0-30.0]	27.0 [17.0-34.0]	0.0469
Adrenaline administered by EMS (N [%])	235 [34.3]	13 [35.1]	222 [34.3]	0.9131
Tracheal intubation performed by EMS (N [%])	21 [3.1]	3 [8.1]	18 [2.8]	0.0985
ECMO attempted (N [%])	37 [5.4]	37 [100.0]	0 [0.0]	NA
ECMO performed (N [%])	36 [5.2]	36 [97.3]	0 [0.0]	NA
Time from hospital arrival until the start of ECMO [min]	29.0 [15.5-37.5]	29.0 [15.8-37.8]	NA	NA
Etiological factors for intrinsic CA				
Cardiac factors (N [%])	440 [64.2]	25 [67.6]	415 [64.0]	0.6636
ACS (N [%])	55 [8.0]	15 [40.5]	40 [6.2]	<0.0001
Non-ACS cardiac disease (N [%])	82 [12.0]	7 [18.9]	75 [11.6]	0.1906
PCF (N [%])	302 [44.1]	3 [8.1]	299 [46.1]	<0.0001
Noncardiac factors (N [%])	245 [35.8]	21 [32.4]	233 [36.0]	0.6636
Respiratory disease (N [%])	54 [7.9]	3 [8.1]	51 [7.9]	1
Cerebrovascular disease (N [%])	39 [5.7]	3 [8.1]	36 [5.6]	0.4608
Malignancy (N [%])	29 [4.2]	0 [0.0]	29 [4.5]	0.395
Other noncardiac disease (N [%])	123 [18.0]	6 [16.2]	117 [18.1]	1
30 days survival (N [%])	37 [5.4]	7 [18.9]	30 [4.7]	0.0024
CPC = 1; 2 after 30 days (N [%])	16 [2.4]	2 [5.6]	14 [2.2]	0.208
90 days survival (N [%])	22 [3.3]	4 [11.1]	18 [2.8]	<0.0001
CPC = 1; 2 after 90 days (N [%])	14 [2.1]	4 [11.1]	10 [1.6]	0.0048

**Figure 3:**
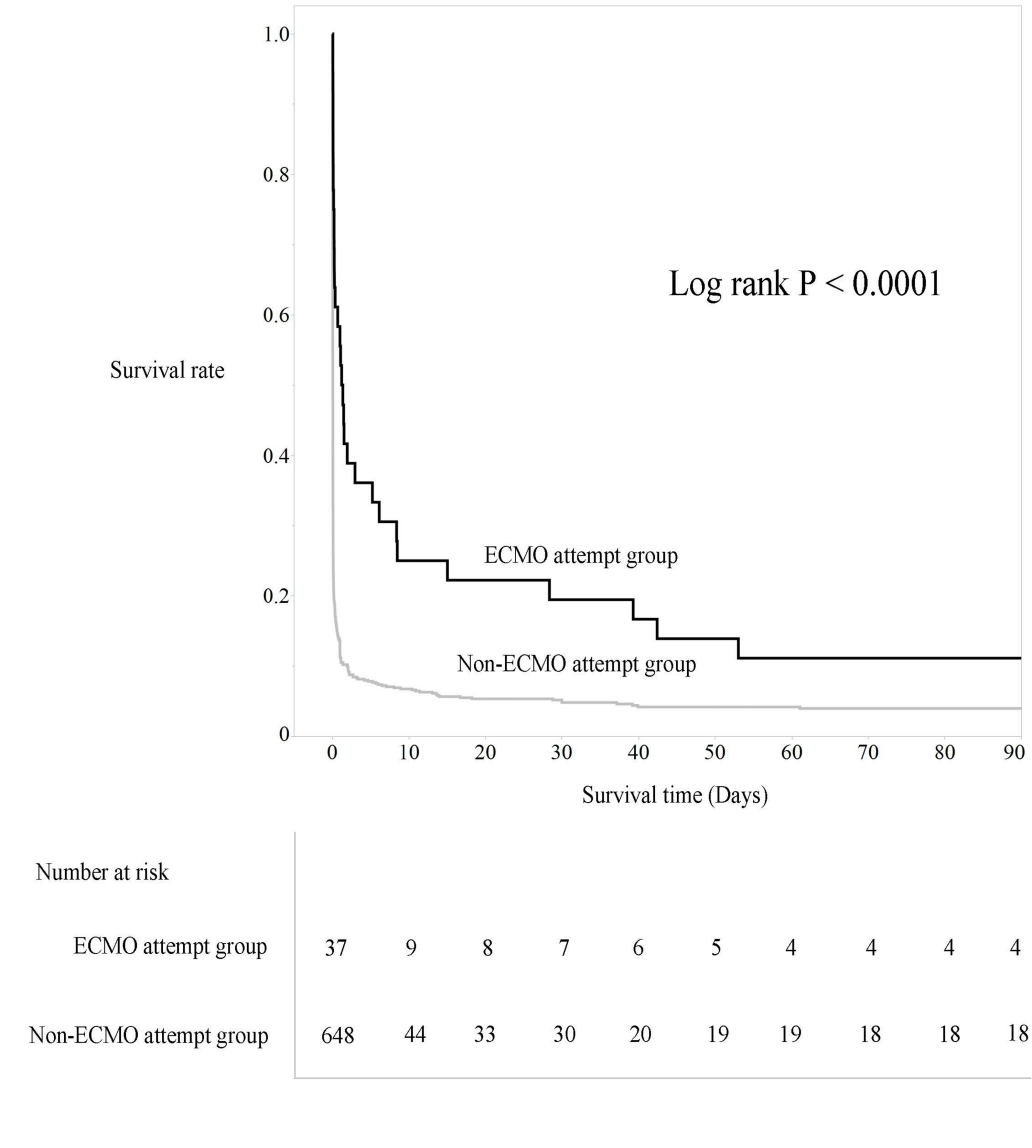
Comparison of survival rates based on the Kaplan-Meier curve in Analysis B Vertical axis: survival rate (%); horizontal axis: survival period (days); blue line: the ECMO attempt group; red line: the non-ECMO attempt group ECMO: extracorporeal membrane oxygenation

As shown in Figure 3, Kaplan-Meier survival curves indicated significantly longer survival up to 90 days in the ECMO attempt group. Regarding favorable neurological outcomes, as shown in Table 3, the percentage of patients with CPC 1-2 at 30 days was 5.6% in the ECMO attempt group and 2.2% in the non-ECMO attempt group, with no significant difference. However, at 90 days, the percentage of patients with CPC 1-2 was significantly higher in the ECMO attempt group.

Additionally, a multivariate model was created to examine favorable neurological outcomes (CPC 1-2) with an ECMO attempt and various explanatory variables. However, since there were only 14 cases with favorable neurological outcomes, it was only possible to include two explanatory variables in a single multivariate model, whether an ECMO attempt was made and one other variable. Therefore, we combined each explanatory variable listed in Table 3 with the ECMO attempt and selected three models that were both medically plausible and had the highest R-squared values. As shown in Table 4, the combination of an ECMO attempt and the time from witnessing CA to hospital arrival had the highest R squared (0.2039) and was considered the best predictive model for favorable neurological outcomes (CPC 1-2). In this context, an ECMO attempt was an independent prognostic factor. However, in a model including ACS, R squared was 0.093, indicating a poorer fit, although an ECMO attempt remained an independent prognostic factor and ACS was an independent prognostic factor.

**Table 4:** Multivariate analyses: favorable cerebral function outcome (CPC = 1/2) at 90 days in Analysis B ACS: acute coronary syndrome; CPC: cerebral performance category; ECMO: extracorporeal membrane oxygenation; PCF: presumed cardiac factor

	OR	95% CI	P	R squared
ECMO attempted	6.259	1.736-22.57	0.0051	0.2039
Time from a witness of CA until hospital arrival (Δ = 1 min)	0.998	0.997-0.999	<0.0001	
ECMO attempted	4.483	1.323-15.19	0.016	0.1591
PCF	<0.001	NA	0.9852	
ECMO attempted	3.919	0.964-15.94	0.0563	0.093
ACS	4.623	1.253-17.05	0.0215	

## Discussion

In the present study, ECPR was associated with not only survival, but also improved neurological outcomes, even for NSOHCA, and this relationship was significant when bystander CPR was provided promptly and the time from CA to hospital arrival was within 45 minutes, with a 90-day good neurological outcome being achieved in 11.1% of cases in the ECMO attempt group in contrast to only 1.6% of cases in the non-ECMO attempt group (p = 0.0048). Even in Analysis B, the time from witnessing CA to hospital arrival was significantly shorter in the ECMO attempt group, and the multivariate analysis indicated that the time from witnessing CA to hospital arrival had a marked impact on favorable neurological outcomes. We previously reported that the time to hospital arrival significantly affected the outcomes of NSOHCA cases with and without ECPR after EMS contact [8]. The present results are consistent with these findings, suggesting that the success of ECPR in NSOHCA, particularly neurological outcomes, was closely related to the time from the onset of CA to the initiation of ECMO.

However, the neurological good outcome rate of 11.1% in the ECMO attempt group in Analysis B for NSOHCA was lower than the reported neurological good outcome rate of between 12.4 and 40.0% with ECPR for SOHCA [1-3]. Since the goal of initiating ECPR within one hour of witnessing CA has been generalized for SOHCA [9-13], there may be fundamental differences in the response to ECPR between SOHCA and NSOHCA beyond the time from witnessing CA to the initiation of ECMO. In studies focusing on SOHCA, both RCTs and observational studies have clearly demonstrated the efficacy of ECPR [2,3,12]. However, in studies that included both SOHCA and NSOHCA, mainly observational studies, the efficacy of ECPR has not always been clear [1,4,5]. In our observational study focusing solely on NSOHCA, we suggested the potential efficacy of ECPR and also emphasized the importance of the time from CA onset to hospital arrival [8], but no other significant factors were identified. Additionally, other studies have not shown significant differences in background factors other than time [1-5]. Among the background factors compared in the present study, no significant difference was observed in the cardiac or non-cardiac etiologies of CA. However, ACS was significantly more common in the ECMO attempt group, whereas a presumed cardiac etiology was significantly less common. The multivariate analysis indicated that in addition to the presence or absence of an ECMO attempt and the time from witnessing CA to hospital arrival, the presence of ACS was associated with neurological outcomes in Analysis A and Analysis B. In the Arrest trial, which reported favorable outcomes with ECPR, seven out of 15 cases undergoing ECMO were treated for coronary artery lesions, in contrast to only two out of 15 non-ECPR cases [3]. Additionally, Sakamoto et al. reported that ACS accounted for 63.5% of ECPR cases and 59.3% of non-ECPR cases in SOHCA [2], while Inoue et al. showed that ACS accounted for 59.0% of ECMO cases [13]. These findings suggest that the lower percentage of ACS as the etiology in NSOHCA than in SOHCA contributed to a better prognosis in the ECMO attempt group than in the non-ECMO attempt group, but not to the extent observed for SOHCA.

A strength of the present study is that it was based on a stratified analysis of a registry of numerous CA cases in the SOS-KANTO 2017 study, allowing for the accumulation of a significant number of cases, even for rare events, such as ECPR with ECMO, at individual hospitals. However, a limitation is the low number of cases with favorable neurological outcomes, which affected our ability to perform propensity score matching and restricted the number of explanatory variables in the multivariate analysis. In the future, larger observational studies or RCTs with an increased number of patients will be needed to verify the efficacy of ECPR for NOSOHCA.

Additionally, as a future direction, the potential for improving ECPR itself through technological innovation should be considered. For example, enhancing the performance of blood pumps used in ECPR to allow the insertion of thinner catheters while maintaining high blood flow could simplify the ECPR approach. This could expand the indications for ECPR, particularly in pre-hospital settings, by reducing the time from witnessing CA to initiating ECPR, ultimately improving patient outcomes.

Limitations

Since this was a retrospective observational study, it is not possible to infer causality. Furthermore, although this was a large-scale, multi-center observational study with nearly 10,000 cases, the number of successful resuscitations in NSOHCA, particularly those with favorable outcomes following ECPR, was low, making a stratified analysis difficult and limiting the number of explanatory variables in the multivariate analysis. In addition, based on previous research, NSOHCA is already considered to have poorer outcomes than SOHCA, which may lead to the avoidance of aggressive treatments, including ECPR, resulting in a lower frequency of its application and greater challenges to achieving favorable results. This may be particularly pronounced in patients with inherently poor prognoses, such as those with old age, decreased physical fitness, or diminished activities of daily living, potentially leading to selection bias. However, it is important to note that the use of V-A ECMO imposes significant physical stress and consumes considerable medical resources, so for cases where a poor prognosis is already anticipated, non-implementation of ECPR may be considered from both ethical and economic perspectives. Additionally, depending on the circumstances of the CA, bystander CPR may not be adequately performed, which could influence the decision of whether to initiate ECPR. Specifically, in a large percentage of OHCA cases, particularly those due to CA of cardiac origin, it is not possible to investigate the cause of CA without achieving ROSC or performing ECPR, which may lead to an underestimation of the rate of ACS in the non-ECMO attempt group. Moreover, in V-A ECMO used for ECPR, if left ventricular function is severely impaired, there is a risk of pulmonary congestion due to the cessation of blood flow from the left ventricle to the aorta and thrombosis due to blood stagnation within the left ventricle, given its role of delivering blood to the aorta. Therefore, V-A ECMO often requires left ventricular venting using devices such as IABP or Impella®; however, the SOS-KANTO 2017 study lacks data on this aspect. Another limitation involves post-CA temperature management therapy being a critical factor for neurological outcomes. According to the 2015 resuscitation guidelines, according to which the resuscitation protocol was performed during the study period, temperature management is recommended for resuscitation cases following CA. However, in the present study, only 33 cases received temperature management therapy, rendering a data analysis impossible.

## Conclusions

The prognosis of NSOHCA cases in which CA was witnessed may be improved by ECPR using V-A ECMO, particularly in those on which CPR was initiated within one minute of onset and the patient arrived at the hospital within 45 minutes. Factors associated with a favorable prognosis included a shorter time from onset to hospital arrival and the likelihood of ACS being the cause of CA. The present results suggest the potential of ECPR to improve the survival and 90-day prognosis of NSOHCA, particularly when bystander CPR is initiated quickly and hospital arrival is prompt.

In the future, it may be necessary to increase the total number of analyses by combining registries and focusing ECPR implementation on subgroups for which it is more likely to be effective through a stratified analysis. Additionally, in order to conduct an evaluation that minimizes or eliminates potential biases considered in this study, a randomized controlled trial for evaluating ECPR in NSOHCA or improvements in the equipment used for ECPR could be considered as future directions.

## Appendices

Appendix A: SOS-KANTO 2017 Steering Council

Tokyo Women’s Medical University (Munekazu Takeda), Kimitsu Chuo Hospital (Nobuya Kitamura), Chiba University Hospital (Taka-aki Nakada), The University of Tokyo (Hideo Yasunaga, Shotaro Aso), Nippon Medical School Musashikosugi Hospital (Takashi Tagami), Chiba Kaihin Municipal Hospital (Yosuke Honma, Yoshihisa Tateishi), Nippon Medical School Hospital (Tomoko Ogasawara), Keio University Hospital (Kei Hayashida), Tokyo Bay Urayasu/Ichikawa Medical Center (Hiraku Funakoshi), Juntendo University Nerima Hospital (Tomohisa Nomura), Tokyo Dental College Ichikawa General Hospital (Masaru Suzuki), Tokyo Metropolitan Bokutoh Hospital (Kazuhiro Sugiyama), Nihon University Hospital (Atsushi Sakurai)

Appendix B: SOS-KANTO 2017 Study Group

Tokai University School of Medicine (Yoshihide Nakagawa); St. Marianna University School of Medicine, Yokohama Seibu Hospital (Minoru Yoshida); Saitama Medical Center Department of Emergency Medicine(ER) (Masaki Hisamura); Kawasaki Municipal Hospital Emergency and Critical Care Center (Kunio Kanao); Japanese Red Cross Maebashi Hospital (Jun Maruyama); Juntendo University Urayasu Hospital (Tadashi Ishihara); Tokyo Women’s Medical University Hospital (Munekazu Takeda); Kimitsu Chuo Hospital (Nobuya Kitamura); Chiba University Graduate School of Medicine (Taku Oshima); Dokkyo Medical University Saitama Medical Center (Daisuke Sugimoto); National Disaster Medical Center (Mayuko Kaneko); Nihon University Hospital (Atsushi Sakurai); Nippon Medical School Tamanagayama Hospital (Chie Tanaka); Tokyo Women’s Medical University Yachiyo Medical Center (Tomohito Sadahiro); Japanese Red Cross Medical Center (Yuta Moroe); National Hospital Organization Mito Medical Center (Yusuke Tsutsumi); Tokyo Metropolitan Tama Medical Center (Tomohide Koyama); Gunma University Graduate School of Medicine (Kazunori Fukushima MD); Saitama Red Cross Hospital (Kazuya Kiyota); Tokyo Metropolitan Bokutoh Hospital (Kazuhiro Sugiyama); Keio University Hospital (Ryo Yamamoto); Teikyo University School of Medicine (Ryuichi Nishi); National Center for Global Health and Medicine Hospital (Makiko Yamamoto); Medical Hospital of Tokyo Medical and Dental University (Naoshi Urushibata); Juntendo University Nerima Hospital (Hiroki Takami); Nihon University Itabashi Hospital (Nami Sawada); National Center for Child Health and Development (Shima Ohnishi); Chiba Aoba Municipal Hospital (Shunsuke Otani); Matsudo City Hospital (Masayuki Yagi); Japanese Red Cross Narita Hospital (Yoshihisa Tateishi); Tokyo Bay Urayasu/Ichikawa Medical Center (Yosuke Honma); OTA Memorial Hospital (Kazuki Akieda); Tokyo Dental College Ichikawa General Hospital (Masaru Suzuki) ; Tokyo The Jikei University Kashiwa Hospital (Izumu Hasegawa); Jichi Medical University Saitama Medical Center (Masahiro Kashiura MD); IUHW Narita Hospital (Ryuhei Igeta); University of Tsukuba Hospital (Yasuaki Koyama MD); Saiseikai Utsunomiya Hospital (Takayuki Ogura); Seirei Hamamatsu General Hospital (Takahiro Atsumi); Seirei Mikatahara General Hospital (Go Makishi); Eastern Chiba Medical Center (Tomoaki Hashida); Nagoya University Hospital (Yuma Yasuda) (http://www.jaam- kanto.jp/sos_kanto/sos_kanto2017_contributors.html)
